# Precautions Taken by Nurses about the Prevention of Hospital-Acquired Infections in Intensive Care Units

**DOI:** 10.12669/pjms.342.14610

**Published:** 2018

**Authors:** Inci Kirtil, Nuray Akyuz

**Affiliations:** 1 Inci Kirtil, M.Sc. Research Assistant, Marmara University Faculty of Health Sciences, Department of Surgical Nursing, Başıbüyük Mah. Maltepe Başıbüyük Yolu Sk. Sağlık Bilimleri Kampüsü No:9/4/1 Maltepe, Istanbul, Turkey; 2 Nuray Akyuz, RN, PhD. Associate Professor, Istanbul University Florence Nightingale, Faculty of Nursing Abide-i Hürriyet Cad. 34381 Şişli, Istanbul, Turkey

**Keywords:** Hospital-acquired infections, Intensive care nurse, Intensive care unit

## Abstract

**Objective:**

To determine the precautions that nurses take for avoiding hospital-acquired infections in intensive care units of a State University Medical Faculty Hospital in Istanbul.

**Methods:**

The research data were collected by a questionnaire developed by the authors. The study was conducted in intensive care units of a medical faculty hospital of a state university in Istanbul province. 85 nurses working in different various intensive care units and providing informed consent participated in the study.

**Results:**

Intravenous catheterization, urinary catheterization, ventilator-associated infections and surgical site infections were assessed. The questionnaire was scored by applying a conversion of 100 to the total scores obtained, with the highest score being 100 and lowest score being 0. The percentage of nurses that practised all of the approaches about preventing hospital-acquired infections was estimated to be 8.2% for catheter-related bloodstream infections, 67.1% for surgical site infections, 72.9% for catheter-associated urinary tract infections, 27.1% for ventilator-associated infections, 29.4% for isolation preventions and 62.5% for attempts related to sterilization/disinfection of the medical devices.

**Conclusion:**

It was seen that nurses use most of the effective measures in order to prevent hospital-acquired infections. The guidelines generated for intensive care units should be updated according to international standards as needed. These guidelines should be used effectively; the differences between intensive care units should be resolved and all nurses should be trained at certain intervals.

## INTRODUCTION

Hospital-Acquired Infections (HAI) are defined as infections which are not present and/or incubating at the time of admission to a hospital or healthcare institution and occur during the process of care and treatment or even after discharge.^1^-^3^ HAI are one of the most frequently-encountered adverse events in providing care and constitute a major public health issue that impacts morbidity, mortality and quality of life.^1^,^2^,^4^,^5^ There are many patient and institution-related factors responsible for the high rates of HAI in intensive care units. Patient-related factors include patient’s age, immunity status, chronic illnesses, nutritional status, medications (especially antibiotics) and exposure to catheterization procedures. On the other hand, institution-related factors include high number of patients receiving care in spite of inadequate number of healthcare workers, the architectural structure of the unit, underestimating hand hygiene, disinfection and sterilization practices and not complying with isolation measures.^5^ The treatment costs and rates of mortality of infections acquired in intensive care units are substantially high. Thus, monitoring and controlling infections is of great importance.^1^,^6^ Intensive care nurses have important duties and responsibilities in this regard.

This study was conducted descriptively in order to determine the precautions that nurses take for avoiding hospital-acquired infections in intensive care units.

## METHODS

The study was conducted in surgical Intensive Care Unit (ICU), emergency ICU, neurosurgery ICU, newborn ICU (NICU), pediatric ICU, coronary ICU, cardiovascular surgery ICU and pediatric surgery intensive care units of a state university medical faculty between April 2011 and June 2011. One hundred thirty six nurses worked in these ICUs, of which 85 nurses who were willing to participate in the study and their informed consents were taken. Data of the study were collected using the questionnaire formed developed in the light of the literature by the researchers after the required approvals (institution and ethical board) were obtained. The first part of the questionnaire included questions regarding the demographic characteristics of the nurses. In the second part, the nurses were asked to check the items that they are practicing, which list what the nurses should consider/do about the prevention of intravenous catheterization, surgical site, urinary catheterization and ventilator-associated infections. It was expected that the nurses were to check all items. The questionnaire was scored by applying a conversion of 100 to the total scores obtained from items that questioned what has to be done for preventing infections, with the highest score being 100 and lowest score being zero.

## RESULTS

When the characteristics of the nurses that participated in the study were reviewed, it was observed that 94.1% were female, 80% had a graduate degree and 28.2% were married. About 42.4% of the nurses had participated in an intensive care unit nursing course.

In this study, the percentage of nurses that practiced measures about preventing hospital-acquired infections was estimated to be 8.2% for catheter-related bloodstream infections, 67.1% for surgical site infections, 72.9% for catheter-associated urinary tract infections and 27.1% for ventilator-associated infections. At the same time, 48.2% reported that “I don’t leave the catheters inserted in non-asepsis in emergency situations on the patient for more than 48 hours; I change it with a new one”, 64.7% reported that “I change peripheral intravenous catheters every 72-96 hours” (Table-I), 96.5% said that “I check the surgical site for signs of infections while changing the dressing” and “I record the color, type and volume of drainage fluid” (Table-II), 96.5% pointed out that “I prevent the contact of the catheter and the catheter entry site with feces” (Table-III), 51.8% stated that “I change the ventilator circuits and moisturizers at least 7 days after the procedure when there is no visible contamination”. All nurses (100%) participating in the current study followed the principles of asepsis during aspiration and other interventions (Table-IV).

**Table-I:** Distribution of practicing interventions for preventing intravenous catheterizationrelated infections

Intravenous Catheterization-Related Infections	n	%
I wash my hands before the procedure	79	92.9
I take barrier measures before the procedure (gloves, sterile drapes, mask etc.)	71	83.5
I absolutely and completely comply with principles of asepsis	79	92.9
I don’t leave the catheters inserted in non-asepsis in emergency situations on the patient for more than 48 hours; I change it with a new one.	41	48.2
Before the procedure I clean the catheter insertion site with 70% alcohol, 10% povidone iodine or 2% chlorhexidine gluconate and wait for it to dry	69	81.2
I don’t need to change infusion sets before 72 hours when there is no suspicion of infections	51	60.0
I change peripheral intravenous catheters every 72-96 hours	55	64.7
I change the catheter dressing immediately when it is moist, compromised or gets dirty, if none is present at least once a week	42	49.4
I assess the catheter insertion site for infections every day	82	96.5
	Min-Max	Ort ±SD
Percentage of Interventions Practiced for Preventing Intravenous Catheterization-Related Infections	11.11-100*	74.38±15.24

* Total score obtained from 9 items that questioned practices that are needed for preventing intravenous catheterization-related infections was converted to 100 and assessed with the highest score being 100 and lowest score being 0.

**Table-II:** Distribution of practicing interventions for preventing surgical-site infections.

Surgical-Site Infections	n	%
I don’t allow the patient to touch her/his incision site	67	78,8
I follow principles of asepsis in every intervention to incision site (drain emptying, changing the dressing etc.)	79	92,9
I check the surgical site for signs of infections while changing the dressing	82	96,5
I record the color, type and volume of drainage fluid	82	96,5
I avoid the drains to go above incision level; I carefully make the drains stay under the incision	72	84,7
	Min-Max	Ort ±SD
Percentage of Interventions Practiced for Preventing Surgical Site Infections	20-100^*^	89.88±17.08

* Total score obtained from 5 items that questioned practices that are needed for preventing surgical site infections was converted to 100 and assessed with the highest score being 100 and lowest score being 0.

**Table-III:** Distribution of practicing interventions for preventing urinary catheterization-related infections.

Urinary Catheterization-Related Infections	n	%
I follow principles of asepsis during catheterization and later interventions	81	95.3
I know that I need to protect the sterility of the equipment to be used	84	98.8
I prevent the movement of the catheter and urethral trauma after catheterization	71	83.5
I protect closed drainage system as long as catheterization continues	77	90.6
I prevent backflow of urine by keeping the urine bag below bladder level	84	98.8
I maintain continuous urine flow by preventing the blockage of catheter	76	89.4
I prevent the contact of the catheter and the catheter entry site with feces	82	96.5
	Min-Max	Ort ±SD
Percentage of Interventions Practiced for Preventing Urinary Catheterization-Related Infections	42.86-100*	94.28±11.06

* Total score obtained from 7 items that questioned practices that are needed for preventing urinary catheterization-related infections was converted to 100 and assessed with the highest score being 100 and lowest score being 0.

**Table-IV:** Distribution of practicing interventions for preventing ventilator-associated infections.

Ventilator-Associated Infections	n	%
I make the patients do deep breathing and coughing exercises after head-neck, thoracic or abdominal operations and make them move in bed	62	72.9
I do patient’s oral care every 4 to 6 hours	71	83.5
I aspirate oropharyngeal secretions when necessary	83	97.6
I keep the bed head of the orally-fed patient at a 30-45º angle	82	96.5
I assess the placement of the nasogastric tube every 4 hours in orally-fed patients	61	71.8
I observe the signs of nutritional intolerance in orally-fed patients (abdominal distention, increased residual volume etc.)	79	92.9
I prevent the patient from lying in supine position continuously; I change the patient’s position every two hours	82	96.5
I change the ventilator circuits and moisturizers at least seven days after the procedure when there is no visible contamination	44	51.8
I prefer disposable equipment if possible; if not, I check their sterility.	80	94.1
I avoid frequent aspirations; I use a new catheter for every aspiration	81	95.3
I follow principles of asepsis during aspiration and other intervention	85	100
I fill the nebulizers (moisturizers) only with sterile water and change them every 24 hours	65	76.5
I prevent the fluid accumulating in the ventilator circuit to flow back to the patient or spilling to the floor	80	94.1
	Min-Max	Ort ±SD
Percentage of Interventions Practiced for Preventing Ventilator-Associated Infections	38.46-100*	86.42±13.09

* Total score obtained from 13 items that questioned practices that are needed for preventing ventilatorassociated infections was converted to 100 and assessed with the highest score being 100 and lowest score being 0.

## DISCUSSION

Evidence-based report supported by Agency for Healthcare Research and Quality suggests that the education level of nurses is correlated with the incidence of error occurring in patients and highlights the importance of nurses having sufficient scientific knowledge and skills and being able to put them into practice.^7^ In this study, we found no statistically significant difference between the education status of the nurses and rates of practicing interventions for intravenous catheterization-associated infections, urinary catheterization-associated infections and ventilator-associated infections (p> 0.05).

Measures and recommendation guidelines published by World Health Organization (WHO) and Society for Healthcare Epidemiology of America significantly emphasize the necessity of hand hygiene.^8^,^9^ Hand hygiene is the most effective method in preventing the transmission of pathogens while providing health care.^9^-^13^ One study on 243 nurses in Iran reported that only 29.6% of the nurses practice hand hygiene in accordance with the “five moments for hand hygiene” rule of WHO.^4^ It was stated in another study that 70% of the nurses reported that hand hygiene is the most important measure in preventing disease transmission.^14^ In the current study, 92.9% of the nurses replied “*I wash my hands before the procedure*” and “*I wash my hands before contacting the patient, I wear gloves and I wash my hands again after the procedure*” (Table-I). It is heartening to note that almost all nurses pay attention to and practice hand hygiene, which has major importance in preventing HAI.

Guidelines published by Centers for Disease Control and Prevention point out that renewing peripheral intravenous catheters every 72 to 96 hours decrease the risk of phlebitis and bacteriemia and catheters that are not inserted in asepsis due to emergency should be renewed within 48 hours in compliance with asepsis principles.^15^ According to another study which reviewed seven different studies that evaluates a total of 4895 patients concluded that there is no significant difference in terms of infections between changing the catheter routinely and changing only when required.^16^ One study reported that there were no complications in peripheral catheters that stayed for more than 96 hours.^17^ In this study, we found that 64.7% of the nurses change peripheral intravenous catheters every 72 to 96 hours (Table-I). This is not compatible with the current literature may be due to most patients treated in the intensive care units having central venous lines and nurses, who work in these units, having heavy work load.

After surgical interventions, the site of incision should be closely monitored for signs of infection and if present the color, smell and volume of the drainage fluid should be recorded.^18^,^19^ Similarly, a study conducted in nurses found that almost all nurses observed the surgical site for signs and symptoms of infection.^20^ In this study, 96.5% of the nurses replied “*I check the surgical site for signs of infections while changing the dressing*” (Table-II). However, in the institution where the study was conducted, changing the incision dressing is performed by physicians and nurses only assist when needed.

Perianal area should be kept clean because feces, exudates or debris accumulate in catheter insertion site due to the properties of perianal area and make up a medium for microorganisms.^18^,^21^ In this study, 96.5% of the nurses replied “*I prevent the contact of the catheter and the catheter entry site with feces*” (Table-III). This result, implying that nurses respond as expected to catheter care, is pleasing.

In a systematic review and meta-analysis study about the effect of changing ventilator circuits in preventing ventilator-associated pneumonia concluded that routine change of ventilator circuits is not necessary and changing only when contaminated or damaged is adequate.^22^ In another study conducted in a pediatric ICU there was no difference between changing ventilator circuit every three days or every seven days in terms of risk of ventilator-associated pneumonia.^23^ In this study, the ratio of nurses replying “*I change the ventilator circuits and moisturizers at least seven days after the procedure when there is no visible contamination*” was 51.8% (Table-IV). This low ratio may be due to institutions having different directives for when and how frequent the ventilator circuits and moisturizers should be changed.

In yet another study which evaluated the effectiveness of ventilator-associated pneumonia care package in preventing ventilator-associated pneumonia found that the care package, which includes asepsis principles during aspiration, decreases the risk of ventilator-associated pneumonia.^24^ All nurses (100%) participating in the current study followed the principles of asepsis during aspiration and other interventions. This shows that nurses adapt completely to asepsis comparable to the literature data for patients in mechanical ventilation support and are aware of their responsibilities for preventing ventilator-associated pneumonia.

### Limitations of the study

The study data were obtained from the nurses working in intensive care units of a single state university hospital in Istanbul. It is difficult to generalize the results of the study. Further studies with bigger sample size are recommended.

## CONCLUSION

There was no statistically significant difference according to the educational status, participating in intensive care nursing course and receiving education about hospital-acquired infections between nurses in terms of intravenous catheterization-associated infections, surgical site infections, urinary catheterization-associated infections and ventilator-associated infections. The percentage of nurses practicing measures for preventing intravenous catheterization, surgical site, urinary catheterization and ventilator-associated infections were observed to be satisfactory.
